# Supplementary material for: k-mer manifold approximation and projection for visualizing DNA sequences
Source: Genome Res. 2025 May;35(5):1234–46. doi: 10.1101/gr.279458.124 (PMC12047656; doi:10.1101/gr.279458.124)
Supplement: Supplement 8 [file Supplemental_Data_1.zip › Supplemental_Data_1/NRL_TGGAGC20NGA_O_4/NRL_TGGAGC20NGA_O_4_KMAP.pdf]

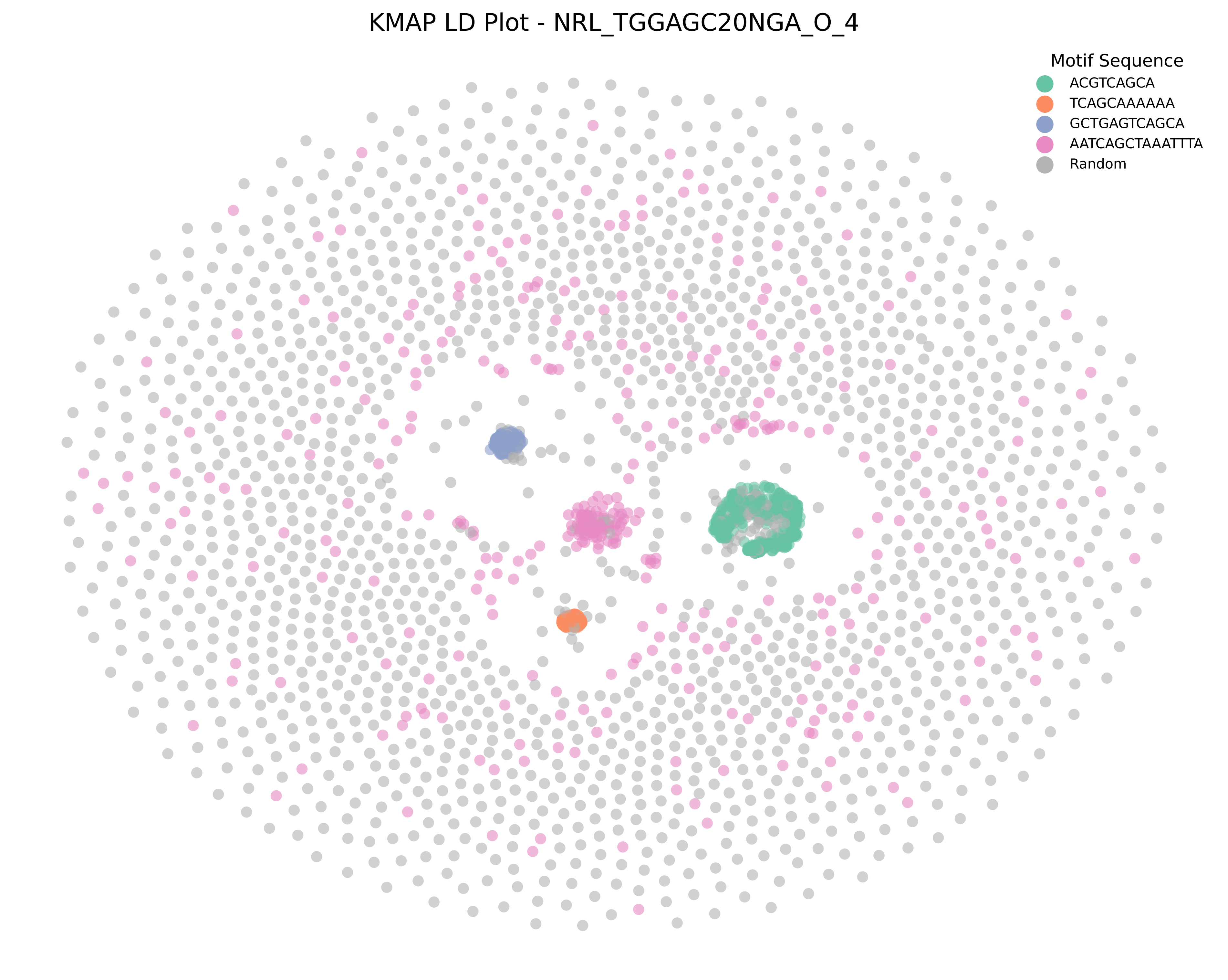

KMAP LD Plot - NRL_TGGAGC20NGA_O_4
Motif Sequence
ACGTCAGCA
TCAGCAAAAAA
GCTGAGTCAGCA
AATCAGCTAAATTTA
Random
